# Containment of a carbapenem-resistant *Klebsiella pneumoniae* in an intensive care unit during the COVID-19 pandemic

**DOI:** 10.3389/fpubh.2025.1557068

**Published:** 2025-06-17

**Authors:** Renhua Li, Zuli Zhang, Zhongjie Wang, Keli Qian

**Affiliations:** ^1^Department of Infection Control, The First Affiliated Hospital of Chongqing Medical University, Chongqing, China; ^2^Department of Respiratory and Critical Care Medicine, The First Affiliated Hospital of Chongqing Medical University, Chongqing, China

**Keywords:** MDRO, CRKP, ICU outbreak, infection control, COVID-19

## Abstract

**Background:**

A nosocomial outbreak of *carbapenem-resistant Klebsiella pneumoniae* (CRKP) occurred in the 20-bed Respiratory Intensive Care Unit (RICU) of a tertiary teaching hospital during the COVID-19 pandemic (December 2022–February 2023). The outbreak was ultimately mitigated through multimodal infection control interventions aligned with WHO multidrug-resistant organism (MDRO) management guidelines.

**Methods:**

Following index case identification on 10 December 2022, a multidisciplinary outbreak response team implemented comprehensive control measures: Immediate geographic cohorting of CRKP-positive patients with dedicated staff; Enhanced contact precautions including daily chlorhexidine bathing; Tri-daily environmental decontamination using sporicidal agents; Mandatory hand hygiene audits with real-time feedback; Active surveillance through weekly rectal swabs for all RICU admissions. Environmental monitoring encompassed 120 high-touch surfaces sampled weekly.

**Results:**

Among 42 laboratory-confirmed CRKP cases, 85.7% (*n* = 36) were identified through clinical specimens and 14.3% (*n* = 6) via active surveillance. Post-outbreak surveillance revealed two imported CRKP cases detected through admission screening during the three-month follow-up period, both contained without secondary transmission. The increasing patient volume, prolonged use of personal protective equipment (PPE), and influx of new healthcare workers heightened the risk of CRKP transmission. Effective administrative guidance on nosocomial infections, behavioral control, active surveillance culture, environmental cleanliness and antimicrobial management are essential to prevent outbreak.

**Conclusion:**

This outbreak demonstrates the viability of containing CRKP transmission in resource-constrained pandemic settings through: rigorous adherence to contact precautions; prospective CRE active surveillance cultures. It is also need to implement antimicrobial stewardship programs in order to reduce the occurrence of microbial resistance.

## Introduction

The escalating global prevalence of antimicrobial resistance (AMR) has been classified by the World Health Organization as a critical public health emergency requiring coordinated international action (1). *Klebsiella pneumoniae* – a Gram-negative Enterobacteriaceae species – demonstrates particular clinical significance due to its propensity to cause nosocomial pneumonia, bloodstream infections, and complicated intra-abdominal syndromes. Carbapenems, historically considered the final therapeutic option for Gram-negative infections, have seen their efficacy undermined by the rapid global dissemination of carbapenem-resistant *K. pneumoniae* (CRKP) clones (2, 3). According to the data from the Institute for Clinical and Laboratory Standards, CRKP are all isolates of *Klebsiella pneumoniae* and are resistant to any carbapenem drugs: Imipenem, or ertapenem. CRKP was resistant to most of the currently available antibiotics, posing a huge threat to human health (4). Surveillance data from the China Antimicrobial Surveillance Network (CHINET) reveal alarming resistance trends: imipenem resistance rates in *K. pneumoniae* isolates surged from 4.9% (2009) to 20.4% (2022), with meropenem resistance paralleling this trajectory (4.8 to 21.9%)^1^. In 2020, the rate of carbapenem resistance for *K. pneumoniae* isolates was exceeding 50% in parts of the Europe and Eastern Mediterranean (5). A recent meta-analysis shows that the incidence rate of CRKP colonization worldwide ranges from 2 to 73%, and the total incidence rate is 22.3% (6). CRKP infections are characterized by limited therapeutic options and substantial mortality burdens, demonstrating 28-day attributable mortality rates of 30–70% across multiple epidemiological studies (7).

The CRKP’s capacity for prolonged environmental persistence (≥12 months on dry surfaces) facilitates nosocomial transmission, particularly in critical care settings (8). Molecular epidemiological investigations identify two primary transmission pathway: direct patient-to-patient spread via healthcare worker hand carriage [accounting for 68% of transmissions in ICU settings and environmental reservoir-mediated infections (9, 10)]. Independent risk factors for CRKP acquisition include prolonged ICU stays, invasive device utilization, and cumulative antibiotic exposure exceeding 14 days (11).

The COVID-19 pandemic has exacerbated AMR progression through multifactorial mechanisms: inappropriate antimicrobial prescribing rates exceeding 70% in COVID-19 management; PPE shortages compromising standard infection control practices; immune dysregulation in critically ill patients increasing vulnerability to MDRO colonization (12–16).

This outbreak investigation details the epidemiological and molecular characteristics of a CRKP cluster (*n* = 42 cases) within the RICU of a Chongqing tertiary teaching hospital during the Omicron variant surge (December 2022–February 2023). We analyze the operational challenges of maintaining infection prevention protocols under pandemic resource constraints, evaluate the effectiveness of implemented containment strategies (including spatial cohorting and hydrogen peroxide vapor decontamination), and propose evidence-based recommendations for mitigating future MDRO outbreaks in post-pandemic critical care environments.

## Methods

### Study setting

The study was conducted at a 3,200-bed tertiary teaching hospital in Southwest China. The outbreak epicenter was the Respiratory Intensive Care Unit (RICU), comprising 2 double-occupancy isolation rooms and a 16-bed open ward. During the COVID-19 pandemic period, this unit functioned as a designated critical care area for managing COVID-19 patients requiring mechanical ventilation. All healthcare workers adhered to enhanced PPE protocols including N95 respirators, gloves, and disposable gowns, with mandatory competency assessments conducted through simulated donning/doffing procedures.

### Surveillance and microbiological methods

For patients presenting clinically suspected infections, microbiological analysis was performed on blood, sputum, and bronchoalveolar lavage fluid (BALF) specimens following standardized collection protocols. Asymptomatic admissions underwent active surveillance through rectal swab screening for CRKP within 24 h of admission. Thereafter, reexaminations were conducted once a week during hospitalization until the patients were discharged or CRKP infection occurred. Bacterial identification and antimicrobial susceptibility testing were performed using the VITEK 2 Compact system (bioMérieux, France) with GN-ID cards. Carbapenemase production was phenotypically confirmed through the modified carbapenem inactivation method (mCIM), with ertapenem (ETP), imipenem (IPM), and meropenem (MEM) minimum inhibitory concentrations (MICs) determined via CLSI-approved broth microdilution (CLSI M100-Ed32, 2022) using *Escherichia coli* ATCC 25922 as the quality control strain.

Due to pandemic-related supply chain disruptions, molecular characterization (including PFGE, whole-genome sequencing, and resistance gene PCR amplification) could not be performed on CRKP isolates, limiting phylogenetic analysis (17).

Environmental surveillance encompassed weekly sampling of: (1) healthcare worker hand surfaces (*n* = 20 random samples/month) using neutralizer-containing transport media; (2) high-touch surfaces in patient zones (bed rails, monitors, ventilator interfaces, oxygen regulators) using pre-moistened sterile swabs (*n* = 100 random samples/month). According to “Regulation of disinfection technique in healthcare settings” (18), common nutrient AGAR plates were used for culture. After the suspicious colonies were isolated, the VITEK2 Compact system (bioMérieux, France) was used for strain determination.

### Ethics statement

The Institutional Review Board at The Frist Affiliated Hospital of Chongqing Medical University approved the present study. The ethics committee granted an exemption for informed consent given the retrospective nature of infection control quality improvement data.

### Statistical analysis

Data were analyzed using SPSS software (version 22.0; IBM Corp., Armonk, NY, United States). Categorical variables are presented as number (n) with percentage (%). Chi-square test were used to evaluate the data. *p* < 0.05 was considered statistically significant.

## Results

### Outbreak description

The index case of the outbreak involved an 88-year-old male transferred to the Respiratory Intensive Care Unit (RICU) on 10 December 2022 with hospital-acquired pneumonia from an external facility. Initial sputum culture obtained at admission revealed CRKP through automated broth microdilution testing 5 days post-collection. Immediate implementation of contact precautions (single-room isolation with dedicated care team) occurred upon microbiological confirmation. Subsequent bronchoalveolar lavage fluid (BALF) analysis from a patient hospitalized since 25 October 2022 demonstrated identical antimicrobial resistance patterns, confirming nosocomial transmission within 48 h of the index case identification.

Following these two initial CRKP identifications, enhanced infection control measures were instituted including: (1) twice-daily chlorhexidine environmental decontamination protocols; (2) cohorting of colonized patients; (3) implementation of dedicated medical equipment for CRKP-positive cases. Despite these interventions, surveillance cultures from two additional patients’ respiratory specimens (sputum/BALF) yielded CRKP with matching resistance profiles 8 days post-intervention initiation. This prompted escalation of control measures: (1) mandatory competency-based hand hygiene training for all RICU staff; (2) spatial segregation of CRKP-positive patients into separate zones; (3) twice-weekly high-touch surface surveillance cultures.

Continuous oversight by hospital infection prevention specialists included real-time observation of 1,284 hand hygiene opportunities by manual log (improvement from 58 to 92% compliance, *p* < 0.001). However, epidemiological surveillance from December 2022 to January 2023 identified 32 laboratory-confirmed CRKP cases through systematic screening of respiratory specimens, blood cultures, and rectal swabs. Concurrent environmental sampling detected CRKP contamination at multiple critical sites: handwashing sink surfaces (45% positivity, 9/20 samples), staff uniforms (30%, 6/20), mobile devices (15%, 3/20), and treatment trays (5%, 1/20). Persistent CRKP detection despite multimodal interventions revealed substantial gaps in environmental disinfection efficacy and equipment sterilization protocols, particularly highlighting contamination persistence in high moisture areas like handwashing sink surfaces.

In February 2023, following persistent detection of CRKP, the Hospital Infection Control Department and RICU conducted a joint evaluation of infection prevention protocols. This collaborative analysis identified critical gaps in existing control measures, prompting implementation of enhanced multimodal interventions. Geographic cohorting was strictly enforced, with all CRKP-positive patients isolated in designated zones staffed by dedicated healthcare teams. Medical equipment including glucometers, portable radiography systems, mechanical ventilators, and continuous renal replacement therapy (CRRT) devices were designated for exclusive use within the CRKP cohort, with rigorous traffic control protocols restricting equipment movement and mandating terminal disinfection between patients. Environmental decontamination was intensified through 24/7 deployment of specialized cleaning teams performing hourly high-touch surface disinfection and tri-daily chlorinated (500 ppm) wipe-downs of all patient care areas. Quality-controlled verification of cleaning efficacy was implemented using adenosine triphosphate bioluminescence monitoring. On February 15, a unit-wide shutdown enabled terminal disinfection utilizing hydrogen peroxide vapor (HPV) technology for airspace decontamination, complemented by sporicidal surface treatment and complete curtain replacement. Post-intervention environmental surveillance on February 16 confirmed eradication of residual contamination (all environmental cultures returned negative results). Subsequent monitoring revealed complete containment with no new CRKP acquisitions detected throughout the remaining surveillance period, demonstrating a statistically significant reduction in transmission rates compared to pre-intervention baseline data. The cohort ultimately included 11 laboratory-confirmed CRKP cases with no secondary transmissions observed.

During the three-month surveillance period from March 1st to June 30th, two cases of CRKP colonization were identified through active surveillance cultures upon hospital admission. Both cases were promptly placed under contact isolation protocols, successfully preventing secondary transmission within the healthcare facility.

**Figure 1:**
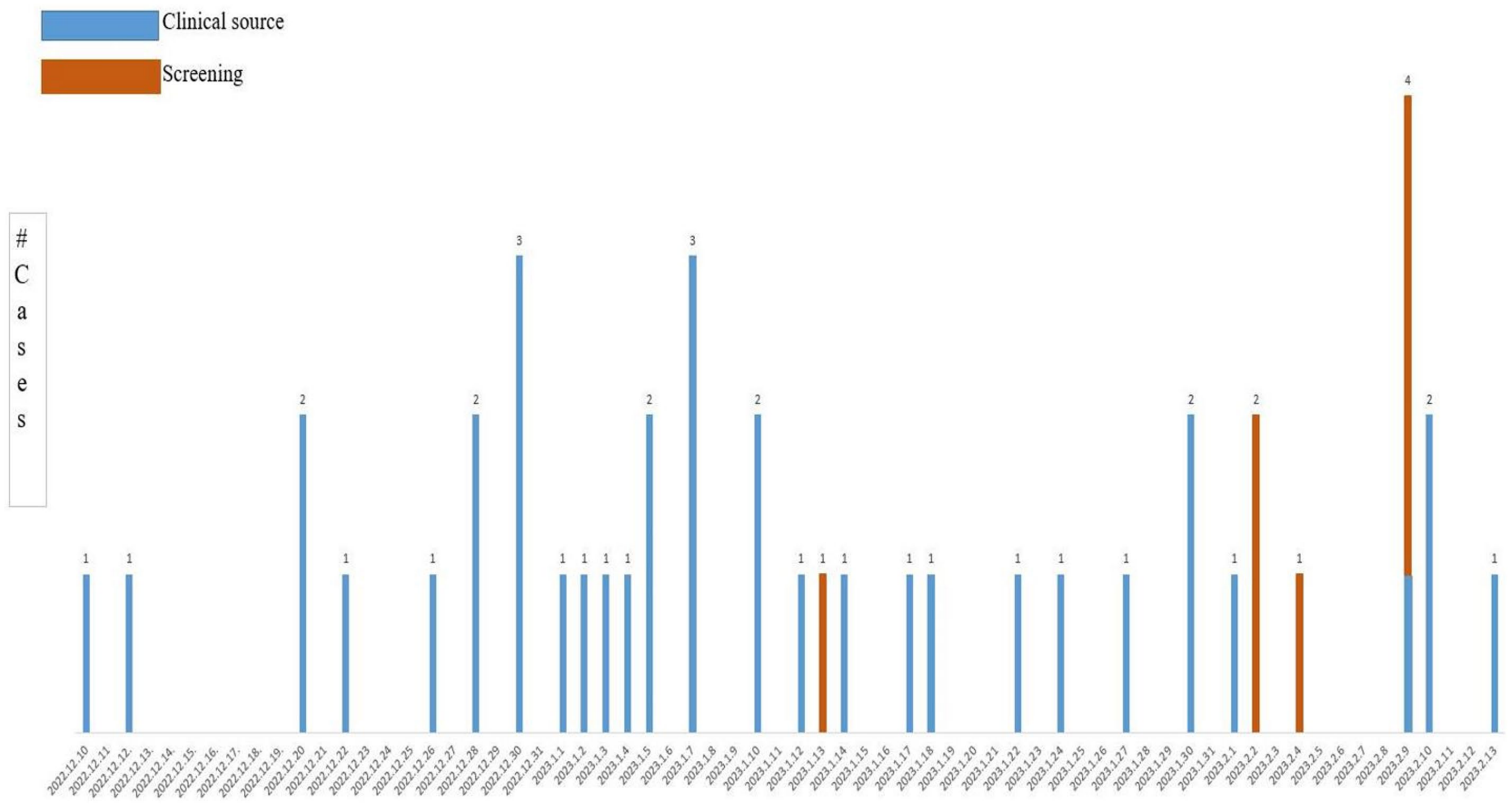
Epidemic curve of CRKP outbreak in RICU. Carbapenem-resistant *Klebsiella pneumoniae* detection time of 42 patients.

Figure 1 chronologically illustrates the CPKP outbreak progression in the Respiratory Intensive Care Unit (RICU), with detailed epidemiological characteristics of the 42 affected patients presented in Supplementary Table S1. Comparative analysis in Table 1 demonstrates a statistically significant reduction in CRKP infection rates when comparing the epidemic phase with the 3-month post-intervention period, indicating the effectiveness of implemented control measures.

**Table 1:** Temporal comparison of CRKP infection rates among RICU hospitalized patients: December 2022 to June 2023.

Period	Total inpatients	CRKP cases	Infection rate (%)	*χ* ^2^	*p*
Dec 1, 2022–Feb 28, 2023	118	42	35.59	47.847	<0.001
Mar 1, 2023–Jun 30, 2023	127	2	1.57		

### Microbiological investigations

Antimicrobial susceptibility testing of carbapenemase-producing *K. pneumoniae* (CPKP) strains isolated from the 42 patients revealed universal resistance to multiple antibiotic classes, with susceptibility retained only to amikacin and gentamicin. All strains tested positive for class A carbapenemase production, confirming their enzymatic resistance mechanism (Supplementary Table S2). Environmental surveillance during the outbreak further identified CPKP contamination across high-touch surfaces, including bedside tables, bed rails, instrument buttons, treatment trays, blood glucose meters, and electrocardiogram equipment. Notably, CPKP was also detected on the hands of cleaning staff, implicating healthcare personnel and environmental reservoirs as critical vectors in transmission (Table 2).

**Table 2:** Positive results of environmental and hand hygiene monitoring in the RICU from December 2022 to March 2023.

Environmental and hand hygiene samples	Dec 2022 (*n*/*N*,%)	Jan 2023 (*n*/*N*,%)	Feb 2023 (*n*/*N*,%)	Mar 2023 (*n*/*N*,%)
Hand hygiene	0/20,0	2/20,10	2/20,10	0/20,0
Handwashing sink surfaces	2/20,10	9/20,45	6/20,30	0/20,0
Staff uniforms	0/20,0	6/20,30	4/20,20	0/20,0
Mobile devices*	0/20,0	3/20,15	5/20,25	0/20,0
Bedside tables and bed rails	0/20,0	2/20,10	4/20,20	0/20,0
Treatment trays	0/20,0	1/20,5	2/20,10	0/20,0

*Mobile devices include electrocardiogram monitors, blood glucose meters, blood pressure monitors, etc.

## Discussion

Multidrug-resistant organisms (MDROs) infections, particularly those caused by *carbapenem-resistant K. pneumoniae* (CRKP), represent a significant challenge in healthcare settings due to their limited treatment options and substantial public health implications. CRKP, recognized as one of the most critical MDRO pathogens, is associated with markedly worse clinical outcomes. Studies indicate that patients with CRKP infections experience prolonged hospitalization, with an average increase of 15.8 days compared to non-CRKP cases. Furthermore, CRKP infections are linked to a mortality rate approximately 2.17 times higher than infections caused by non-resistant strains, underscoring the urgent need for targeted interventions to mitigate its impact (19).

This study investigated a CRKP outbreak involving 42 cases in a tertiary hospital in Chongqing, China. While conventional wisdom posits that enhanced infection control measures during the COVID-19 pandemic would suppress multidrug-resistant organism (MDRO) transmission, our findings align with global reports of paradoxical MDRO surges during this period (20, 21). The contributing factors are multifaceted: firstly, pandemic-driven human resource strain. The abrupt surge of COVID-19 patients necessitated redeployment of healthcare staff, including personnel lacking expertise in infection prevention protocols, to high-risk units such as isolation wards and intensive care units (ICUs). This disruption likely compromised adherence to MDRO containment practices (22). Also, the reduction in human resources has led to a decrease in the supervision of infection control measures. Secondly, pandemic-driven infection control lapses. An overemphasis on COVID-19-specific precautions—such as prioritizing glove use over hand hygiene—may have inadvertently reduced compliance with fundamental infection control measures (23, 24). For instance, improper or prolonged use of personal protective equipment (PPE) without concurrent environmental hygiene protocols created ambiguities in maintaining sterile patient environments. PPE, while protecting healthcare workers, fails to prevent MDRO colonization or transmission to patients if environmental disinfection is neglected. Concurrently, PPE shortages forced facilities to ration or reuse supplies, further escalating cross-contamination risks (25, 26). Thirdly, antibiotic misuse related to the COVID-19 pandemic. The high antibiotic selection pressure due to the high proportion of patients with COVID-19 infection, especially those with severe illness, who are empirical using broad-spectrum antibiotics, contributes to the generation of MDROs (27). Rawson et al. found that up to 72% of COVID-19 patients were given broad-spectrum antibiotics (28). The near-universal empirical use of broad-spectrum antibiotics in our cohort (41/42 patients) highlights a critical vulnerability. Such practices not only fuel resistance but also obscure the clinical distinction between viral and bacterial infections, complicating outbreak management.

Environmental disinfection and infrastructure deficiencies. While our outbreak shares similarities with global MDROs trends—such as staff redeployment, lapses in routine infection control, and antibiotic overuse—distinct differences emerge. For instance, in European hospitals, CRKP outbreaks during the pandemic were frequently linked to overwhelmed intensive care units (ICUs) and prolonged patient stays (29). In contrast, our study identified environmental reservoirs (e.g., sinks, medical equipment) and healthcare worker-mediated transmission as primary drivers, a pattern more commonly reported in resource-limited settings where infrastructure maintenance lags. Epidemiological investigations have identified multiple potential sources of infection during multidrug-resistant organism (MDRO) outbreaks, including colonized patients, contaminated hospital environments (e.g., sinks, toilets), and healthcare workers acting as transient vectors (30–32). During such outbreaks, resistant pathogens frequently colonize high-touch surfaces near patient beds and medical equipment. Notably, certain resilient strains, including CRKP, can persist on dry surfaces for months, facilitating persistent environmental reservoirs (33–36). Transmission dynamics are further compounded by healthcare workers’ roles as carriers, with studies demonstrating that drug-resistant bacteria predominantly spread via contaminated hands, attire, or medical devices (37).

In this outbreak, the spatial separation of the first two patients’ beds—coupled with the detection of CRKP on environmental surfaces (e.g., bed rails, sinks), healthcare workers’ hands, and medical instruments (e.g., electrocardiogram machines)—strongly implicates healthcare personnel or environmental contamination as key transmission routes. Genotypic and phenotypic analyses of bacterial resistance profiles further confirmed epidemiological linkages between patient isolates and environmental samples, definitively classifying this event as a nosocomial outbreak. Following the implementation of stringent infection control measures—including enhanced environmental disinfection, strict hand hygiene protocols, and contact precautions—transmission was effectively curtailed, with no subsequent CRKP cases detected in patients or environmental screenings.

The detection rate of CRKP in medical institutions has reached almost 22% in China. Nosocomial infection outbreaks that can spread rapidly and cause serious consequences if not actively controlled after detection. Therefore, strict infection management measures, such as active surveillance and isolation of positive colonized patients, are required to prevent and respond to the spread of drug-resistant bacteria in hospitals, especially in the ICU. To mitigate future outbreaks, a dual focus on active surveillance and antimicrobial stewardship is imperative. First, proactive CRKP screening, particularly in high-risk populations (e.g., ICU admissions, immunocompromised patients), must be prioritized. Studies demonstrate that active surveillance cultures (ASCs) reduce CRKP transmission by 40–60% when combined with preemptive isolation (38). However, currently in China, ASC implementation remains limited due to cost concerns, leaving facilities vulnerable to undetected colonization. But according to a research report from Maryland, the implementation of a statewide CRE active surveillance and registry reduced annual CRE infections by 6.3% and estimated to save $572,000 statewide in averted infections per year (39). We advocate for national guidelines mandating ASCs in high-risk units, as seen in Italy, early detection and isolation have effectively reduced the spread of CRKP in ICUs (40). Second, antimicrobial stewardship programs (ASPs) must address the pervasive use of broad-spectrum antibiotics. In our cohort, all patients received such agents, mirroring global trends that most COVID-19 patients have been prescribed unnecessary antibiotics (41, 42). ASPs incorporating real-time prescribing audits and clinician education have proven effective in reducing inappropriate antibiotic use, thereby lowering selection pressure for MDROs (43, 44). Following the pandemic, our institution has implemented enhanced antimicrobial stewardship measures through a multidisciplinary ASP framework. As a core intervention, clinical pharmacists now conduct prospective audits of all antimicrobial prescriptions to ensure adherence to evidence-based prescribing protocols. Notably, the prescription of restricted antimicrobial agents (particularly carbapenems and extended-spectrum antibiotics) by non-infectious diseases specialists requires prior authorization following a comprehensive assessment. This authorization process mandates collaborative evaluation by either a board-certified infectious diseases physician or an antimicrobial stewardship clinical pharmacist, who verify the clinical necessity through systematic review of microbial culture results, inflammatory markers, and clinical symptom progression. This tiered authorization system aims to optimize clinical outcomes while mitigating antimicrobial resistance through appropriate spectrum targeting.

## Conclusion

The interplay of pandemic-related disruptions and preexisting gaps in infection control fueled this CRKP outbreak. While short-term measures (e.g., contact precautions, staff training) successfully contained transmission, sustained resilience demands actionable reforms. Specific recommendations for future outbreaks include: (1) mandatory admission screening protocols for high-risk populations (e.g., ICU transfers, immunocompromised patients) using rapid PCR-based assays to enable early isolation; (2) strict enforcement of antibiotic stewardship programs (ASPs), including preauthorization for carbapenems and real-time feedback on prescribing patterns; (3) standardized environmental monitoring to validate cleaning efficacy in critical care units. By institutionalizing such targeted strategies, healthcare systems can preempt transmission chains and mitigate the dual threat of MDROs in both clinical and community settings.

## Data Availability

The original contributions presented in the study are included in the article/Supplementary material, further inquiries can be directed to the corresponding author.

## Footnotes

^1^ http://chinets.com/Data/GermYear
