# Correction: Containment of a carbapenem-resistant *Klebsiella pneumoniae* in an intensive care unit during the COVID-19 pandemic

**DOI:** 10.3389/fpubh.2025.1650017

**Published:** 2025-07-08

**Authors:** Renhua Li, Zuli Zhang, Zhongjie Wang, Keli Qian

**Affiliations:** ^1^Department of Infection Control, The First Affiliated Hospital of Chongqing Medical University, Chongqing, China; ^2^Department of Respiratory and Critical Care Medicine, The First Affiliated Hospital of Chongqing Medical University, Chongqing, China

**Keywords:** MDRO, CRKP, ICU outbreak, infection control, COVID-19

In the published article, there was an error in the equal contribution statement. The corrected author list appears below.

Renhua Li^1†^, Zuli Zhang^2†^, Zhongjie Wang^1^, Keli Qian^1^

^†^These authors share first authorship

The original article has been updated.

