# Supplementary material for: Containment of a carbapenem-resistant Klebsiella pneumoniae in an intensive care unit during the COVID-19 pandemic
Source: Front Public Health. 2025 Jun 17;13:1557068. doi: 10.3389/fpubh.2025.1557068 (PMC12209329; doi:10.3389/fpubh.2025.1557068)
Supplement: Supplementary file 1 [file Supplementary_file_1.pdf]

TableS1 Clinical characteristics of 42 patients with CRKP isolates.

Patient	Age	Sex	ICU admission date	Specimen collection	Specimen	Diagnosis	COVID-19	Medical devices	Outcomes
1	88	M	2022.12.10	2022.12.10	Sputum	Pneumonia	Positive	Noninvasive ventilation, foley catheter	Deceased
2	70	M	2022.10.25	2022.12.12.	BALF	Pneumonia	Positive	Internal jugular vein catheter, Endotracheal tube (ventilator), foley catheter	Cured
3	85	M	2022.12.14	2022.12.20	BALF	Pneumonia	Positive	Internal jugular vein catheter, Endotracheal tube (ventilator), foley catheter	Deceased
4	68	M	2022.10.25	2022.12.20	Sputum	Acute upper gastrointestinal bleeding	Positive	Internal jugular vein catheter, Endotracheal tube (ventilator), foley catheter	Cured
5	76	M	2022.12.12	2022.12.22	Sputum	Pneumonia	Positive	Internal jugular vein catheter, Endotracheal tube (ventilator), foley catheter	Deceased
6	68	F	2022.12.17	2022.12.26	BALF	Pneumonia	Positive	Internal jugular vein catheter, Endotracheal tube (ventilator), foley catheter	Deceased
7	52	M	2022.12.19	2022.12.28	BALF	Pneumonia	Positive	Internal jugular vein catheter, Endotracheal tube (ventilator), foley catheter	Discontinue treatment
8	76	M	2022.12.22	2022.12.28	Sputum	Acute exacerbation	Positive	Internal jugular vein	Deceased

						of chronic obstructive pulmonary disease		catheter, Endotracheal tube (ventilator), foley catheter	
9	86	F	2022.12.24	2022.12.30	Sputum	Pneumonia	Positive	Internal jugular vein catheter, Endotracheal tube (ventilator), foley catheter	Deceased
10	76	M	2022.12.25	2022.12.30	Sputum	Pneumonia	Positive	Internal jugular vein catheter, Endotracheal tube (ventilator), foley catheter	Abandoned
11	65	M	2022.12.22	2022.12.30	Sputum	Pneumonia	Positive	Internal jugular vein catheter, Endotracheal tube (ventilator), foley catheter	Deceased
12	75	M	2023.1.9	2023.1.1	Sputum	Pneumonia	Positive	none	Transfer
13	72	M	2022.12.28	2023.1.2	Sputum	Pneumonia	Positive	Internal jugular vein catheter, Endotracheal tube (ventilator), foley catheter	Discontinue treatment
14	76	M	2022.12.30	2023.1.3	BALF	Pneumonia	Positive	Internal jugular vein catheter, Endotracheal tube (ventilator)	Deceased
15	82	F	2022.12.26	2023.1.4	Blood	Pneumonia	Positive	Internal jugular vein catheter, Endotracheal tube (ventilator), Foley catheter	Deceased
16	75	M	2022.12.22	2023.1.5	Sputum	Pneumonia	Positive	Foley catheter	cured
17	72	M	2023.12.17	2023.1.5	Sputum	Pneumonia	Positive	Endotracheal tube (ventilator), Foley catheter	Transfer
18	91	M	2023.1.3	2023.1.7	Sputum	Pneumonia	Positive	Internal jugular vein	Deceased

								catheter, Foley catheter	
19	85	M	2023.1.4	2023.1.7	Sputum	Pneumonia	Positive	Internal jugular vein catheter, Endotracheal tube (ventilator), Foley catheter	Deceased
20	83	F	2023.1.1	2023.1.7	Sputum	Pneumonia	Positive	Endotracheal tube (ventilator), Foley catheter	Deceased
21	68	M	2022.12.29	2023.1.10	BALF	Pneumonia	Positive	Internal jugular vein catheter, Endotracheal tube (ventilator), Foley catheter	Deceased
22	84	M	2023.1.6	2023.1.10	Blood	Pneumonia	Positive	Internal jugular vein catheter, Endotracheal tube (ventilator), Foley catheter	Deceased
23	66	M	2023.1.9	2023.1.12	Sputum	Pneumonia	Positive	Internal jugular vein catheter, Endotracheal tube (ventilator), Foley catheter	Deceased
24	74	M	2023.1.4	2023.1.13	Rectal swab	Lymphoma	Positive	Noninvasive ventilation, foley catheter	Deceased
25	91	M	2023.1.5	2023.1.14	Sputum	Acute exacerbation of chronic obstructive pulmonary disease	Negitive	Internal jugular vein catheter, Noninvasive ventilation, foley catheter	Deceased
26	83	M	2023.1.4	2023.1.17	Sputum	Pneumonia	Positive	Endotracheal tube (ventilator), Foley catheter	Discontinue treatment
27	70	M	2023.1.12	2023.1.18	Sputum	Pneumonia	Positive	Internal jugular vein catheter, Endotracheal tube (ventilator), Foley catheter	Cured

28	53	M	2023.1.18	2023.1.22	BALF	Pneumonia	Positive	Internal jugular vein catheter	Cured
29	62	M	2022.12.26	2023.1.24	Blood	Pneumonia	Positive	Internal jugular vein catheter, Endotracheal tube (ventilator)	Transfer
30	74	M	2023.1.17	2023.1.27	Sputum	Pneumonia	Positive	Internal jugular vein catheter, Endotracheal tube (ventilator), Foley catheter	Deceased
31	36	M	2023.1.21	2023.1.30	BALF, Blood	Sepsis	Positive	Internal jugular vein catheter, Endotracheal tube (ventilator)	Deceased
32	67	M	2023.1.29	2023.1.30	Sputum	Pneumonia	Positive	Noninvasive ventilation	Deceased
33	58	M	2023.1.24	2023.2.1	Sputum	Pneumonia	Positive	Internal jugular vein catheter, Endotracheal tube (ventilator), Foley catheter	Deceased
34	73	M	2023.1.24	2023.2.2	Rectal swab	Acute exacerbation of chronic obstructive pulmonary disease	Positive	Internal jugular vein catheter, Endotracheal tube (ventilator), Foley catheter	Cured
35	38	M	2023.1.20	2023.2.2	Rectal swab	Pneumonia	Positive	Internal jugular vein catheter, Endotracheal tube (ventilator), Foley catheter	Transfer
36	68	M	2023.2.2	2023.2.4	Rectal swab, Blood	Pneumonia	Positive	Internal jugular vein catheter, Endotracheal tube (ventilator), Foley catheter	Discontinue treatment
37	81	M	2023.1.19	2023.2.9	Sputum	Pneumonia	Positive	Internal jugular vein	Deceased

								catheter, Endotracheal tube (ventilator), Foley catheter	
38	67	F	2023.1.31	2023.2.9	Rectal swab	Pneumonia	Positive	Internal jugular vein catheter, Endotracheal tube (ventilator)	Deceased
39	84	M	2023.2.2	2023.2.9	Rectal swab	Pneumonia	Positive	Internal jugular vein catheter, Endotracheal tube (ventilator)	Cured
40	91	M	2022.9.11	2023.2.9	Rectal swab	Pneumonia	Positive	Internal jugular vein catheter, Endotracheal tube (ventilator), Foley catheter	Cured
41	75	M	2023.2.3	2023.2.10	Sputum	Pneumonia	Positive	Internal jugular vein catheter, Endotracheal tube (ventilator), Foley catheter	Deceased
42	88	M	2023.1.31	2023.2.13	BALF	Pneumonia	Positive	Endotracheal tube (ventilator), Foley catheter	Deceased

TableS2 Antimicrobial susceptibility profile of 42 CRKP isolates.

Patients	Amikacin	Aztreonam	Ceftazidime	Ciprofloxacin	Cefotaxime	Cefuroxime	Cefazolin	Cefepime	Gentamicin	Levofloxacin	Ampicillin/sulbactam	Trimethoprim/sulfamethoxazole	Piperacillin/Tazobactam	Meropenem	Imipenem	Tigecycline	A Enzyme	B Enzyme
1	≥64	6	≥64	6	≥64	≥64	6	6	10	≥8	≥6	≥16/304	≥128	6	≥16	≥8	+	-
2	≥64	6	≥64	6	≥64	≥64	6	6	12	≥8	≥6	≥16/304	≥128	6	≥16	≥8	+	-
3	≥64	6	≥64	6	≥64	≥64	6	≥32	8	≥8	≥6	≥16/304	≥128	6	≥16	1	+	-
4	≥64	6	≥64	6	≥64	≥64	6	≥32	8	≥8	≥6	≥16/304	≥128	6	≥16	≥8	+	-
5	≥64	6	≥64	6	≥64	≥64	6	≥32	8	≥8	≥6	≥16/304	≥128	6	≥16	≥8	+	-
6	≥64	6	≥64	6	≥64	≥64	6	≥32	9	≥8	≥6	≥16/304	≥128	6	≥16	≥8	+	-
7	≥64	6	≥64	6	≥64	≥64	6	≥32	9	≥8	≥6	≥16/304	≥128	6	≥16	≥8	+	-
8	≥64	6	≥64	6	≥64	≥64	6	≥32	8	≥8	≥6	≥16/304	≥128	6	≥16	≥8	+	-
9	≥64	6	≥64	6	≥64	≥64	6	≥32	6	≥8	≥6	≥16/304	≥128	6	≥16	≥8	+	-
10	≥64	6	≥64	6	≥64	≥64	6	≥32	9	≥8	≥6	≥16/304	≥128	6	≥16	≥8	+	-

11	≥64	6	≥64	6	≥64	≥64	6	≥32	10	≥8	≥6	≥16/304	≥128	6	≥16	≥8	+	-
12	≥64	6	≥64	6	≥64	≥64	6	≥32	8	≥8	≥6	≥16/304	≥128	6	≥16	≥8	+	-
13	≥64	6	≥64	6	≥64	≥64	6	≥32	8	≥8	≥6	≥16/304	≥128	6	≥16	≥8	+	-
14	≥64	6	≥64	6	≥64	≥64	6	≥32	8	≥8	≥6	≥16/304	≥128	6	≥16	≥8	+	-
15	≥64	6	≥64	6	≥64	≥64	6	≥32	8	≥8	≥6	≥16/304	≥128	6	≥16	≥8	+	-
16	≥64	6	≥64	6	≥64	≥64	6	≥32	9	≥8	≥6	≥16/304	≥128	6	≥16	≥8	+	-
17	≥64	6	≥64	6	≥64	≥64	6	≥32	8	≥8	≥6	≥16/304	≥128	6	≥16	≥8	+	-
18	≥64	6	≥64	6	≥64	≥64	6	≥32	10	≥8	≥6	≥16/304	≥128	6	≥16	≥8	+	-
19	≥64	6	≥64	6	≥64	≥64	6	≥32	8	≥8	≥6	≥16/304	≥128	6	≥16	≥8	+	-
20	≥64	6	≥64	6	≥64	≥64	6	≥32	8	≥8	≥6	≥16/304	≥128	6	≥16	≥8	+	-
21	≥64	6	≥64	6	≥64	≥64	6	≥32	6	≥8	≥6	≥16/304	≥128	6	≥16	≥8	+	-
22	≥64	6	≥64	6	≥64	≥64	6	≥32	8	≥8	≥6	≥16/304	≥128	6	≥16	≥8	+	-
23	≥64	6	≥64	6	≥64	≥64	6	≥32	8	≥8	≥6	≥16/304	≥128	6	≥16	≥8	+	-

24	≥64	6	≥64	6	≥64	≥64	6	≥32	6	≥8	≥6	≥16/304	≥128	6	≥16	≥8	+	-
25	≥64	6	≥64	6	≥64	≥64	6	≥32	6	≥8	≥6	≥16/304	≥128	6	≥16	≥8	+	-
26	≥64	6	≥64	6	≥64	≥64	6	≥32	8	≥8	≥6	≥16/304	≥128	6	≥16	≥8	+	-
27	≥64	6	≥64	6	≥64	≥64	6	≥32	8	≥8	≥6	≥16/304	≥128	6	≥16	≥8	+	-
28	≥64	6	≥64	6	≥64	≥64	6	≥32	9	≥8	≥6	≥16/304	≥128	6	≥16	≥8	+	-
29	≥64	6	≥64	6	≥64	≥64	6	≥32	6	≥8	≥6	≥16/304	≥128	6	≥16	≥8	+	-
30	≥64	6	≥64	6	≥64	≥64	6	≥32	6	≥8	≥6	≥16/304	≥128	6	≥16	≥8	+	-
31	≥64	6	≥64	6	≥64	≥64	6	≥32	6	≥8	≥6	≥16/304	≥128	6	≥16	≥8	+	-
32	≥64	6	≥64	6	≥64	≥64	6	8	6	≥8	≥6	≥16/304	≥128	6	≥16	≥8	+	-
33	≥64	6	≥64	6	≥64	≥64	6	≥32	8	≥8	≥6	≥16/304	≥128	6	≥16	≥8	+	-
34	≥64	6	≥64	6	≥64	≥64	6	≥32	10	≥8	≥6	≥16/304	≥128	6	≥16	≥8	+	-
35	≥64	6	≥64	6	≥64	≥64	6	≥32	10	≥8	≥6	≥16/304	≥128	6	≥16	≥8	+	-
36	≥64	6	≥64	6	≥64	≥64	6	≥32	10	≥8	≥6	≥16/304	≥128	6	≥16	≥8	+	-

37	≥64	6	≥64	6	≥64	≥64	6	≥32	10	≥8	≥6	≥16/304	≥128	6	≥16	≥8	+	-
38	≥64	6	≥64	6	≥64	≥64	6	≥32	10	≥8	≥6	≥16/304	≥128	6	≥16	≥8	+	-
39	≥64	6	≥64	6	≥64	≥64	6	≥32	6	≥8	≥6	≥16/304	≥128	6	≥16	≥8	+	-
40	≥64	6	≥64	6	≥64	≥64	6	≥32	8	≥8	≥6	≥16/304	≥128	6	≥16	≥8	+	-
41	≥64	6	≥64	6	≥64	≥64	6	≥32	9	≥8	≥6	≥16/304	≥128	6	≥16	≥8	+	-
42	≥64	6	≥64	6	≥64	≥64	6	≥32	10	≥8	≥6	≥16/304	≥128	6	≥16	≥8	+	-
